# Influence of Unequal Amplification of Methylated and Non-Methylated Template on Performance of Pyrosequencing

**DOI:** 10.3390/genes13081418

**Published:** 2022-08-09

**Authors:** Olga Taryma-Lesniak, Tina E. Kjeldsen, Lise Lotte Hansen, Tomasz K. Wojdacz

**Affiliations:** 1 Independent Clinical Epigenetics Laboratory, Pomeranian Medical University in Szczecin, Unii Lubelskiej 1, 71-252 Szczecin, Poland; 2 Department of Biomedicine, Aarhus University, Høegh-Guldbergs Gade 10 Build. 1116, 8000 Aarhus C, Denmark; 3 Aarhus Institute of Advanced Studies, Høegh-Guldbergs Gade 6B, 8000 Aarhus C, Denmark

**Keywords:** pyrosequencing, DNA methylation, IVD, in vitro diagnostic

## Abstract

Pyrosequencing is one of the technologies widely used for quantitative methylation assessment. The protocol of pyrosequencing experiment consists of PCR amplification of a locus of interest and subsequent sequencing via synthesis of the amplified PCR product. As the PCR in this protocol utilizes one primer set for the amplification of a template originating from both methylated and non-methylated versions of the analysed locus, the unequal amplification of one of the templates may affect the methylation level assessment by pyrosequencing. We have investigated whether the unequal amplification of one of the templates challenges the quantitative properties of the pyrosequencing technology. Our results show that the sensitivity and dynamic range of pyrosequencing can be significantly affected by unequal amplification of the methylated and non-methylated version of the locus of interest in an assay specific manner. Thus, the assessment of the effect of unequal template amplification on the performances of the specific pyrosequencing assay is necessary before using the assay either in research or especially in diagnostic settings.

## 1. Introduction

Sodium bisulphite selectively converts non-methylated cytosines into uracil, while 5-methylcytosines are resistant to this modification and remain unchanged [1]. This permits the use of PCR to study the methylation status of cytosines after bisulphite conversion, as the unmethylated cytosines will be deaminated to uracil and replaced with thymine during amplification, whereas methylated cytosines remain unchanged.

Two types of primers, methylation specific PCR primers (MSP) and methylation independent PCR primers (MIP), can be designed to amplify bisulphite-modified templates. The MSP primers selectively amplify only a fully methylated template and, therefore, should contain as many CpG sites as possible in the primer binding site [2]. To confirm non-methylated status of a given locus using the MSP approach, a second set of primers needs to be designed, targeting only the non-methylated version of the locus. These primers should contain thymines within CpG dinucleotides at the primer binding site. This type of primers was first utilized in the methylation-specific PCR (MSP) technology [2] and the quantitative adaptation of this technology; methylation-specific PCR (qMSP) was subsequently developed [3].

The MIP primers are designed to amplify bisulphite-modified templates regardless of methylation status and, therefore, are most frequently designed to target regions devoid of CpG sites. The quantification of the methylation level with technologies relying on MIP primers, such as pyrosequencing assumes equal amplification of methylated and non-methylated versions of the locus of interest. However, the equal amplification of the template originating from the methylated and non-methylated templates can be hampered by the PCR bias phenomenon. The PCR bias in methylation studies was first described by P. M. Warnecke et al. [4] as the preferential amplification of the methylated or non-methylated version of the locus from the bisulphite-modified template and non-methylated version of the locus is most frequently reported as preferentially amplified [5]. Considering that the majority of clinically relevant assays aim to detect hypermethylation of a specific locus in clinical samples, which in large proportion consists of healthy tissue with a non-methylated locus of interest, PCR bias can significantly influence the sensitivity of the methylation detection in clinical materials.

With the increasing use of pyrosequencing, not only in research but more importantly in in vitro diagnostic (IVD) methylation biomarker testing, we evaluated to what extent the unequal amplification of the templates affects the performance of the pyrosequencing assays.

## 2. Methods

### 2.1. Control Template

We assessed the influence of the PCR bias on amplification efficiency using dilutions of methylated in non-methylated templates (EpiTect Control DNA, Qiagen, Hilden, Germany) in the range of ratios including: 0% (100% non-methylated), 1%, 10%, 25%, 50%, 75% (of methylated template in non-methylated background), and 100% methylated template.

### 2.2. The Pyrosequencing Assays

The assays used in this study targeted promoters of *RUNX3*, *ABCB1*, *TRIM58*, and *TNF1* genes and were previously published. The details of the assay designs are shown in Table 1. We also used one commercially available assay, therascreen^®^ MGMT Pyro^®^ Kit (Qiagen Gmbh, Hilden, Germany), targeting exon 1 of *MGMT* gene.

The pre pyrosequencing PCRs contained 10× PCR Buffer, 1.6 mM MgCl_2_, dNTP mix (0.4 mM of each), 1U of HotStarTaq DNA Polymerase (QIAGEN GmbH, Hilden, Germany), 200 nM of each primer, and 200–500 ng input DNA. The PCR amplifications were performed as in the original publications (Table 1) for *RUNX3*, *ABCB1*, *TRIM58*, and *TNF1* genes and as recommended by the manufacturer for the *MGMT* gene. Pyrosequencing of the amplified PCR product was carried out using the PyroMark^TM^ Q24 instrument (QIAGEN GmbH, Hilden, Germany) according to the manufacturer protocol. All the experiments were repeated at least three times for each of the analysed sequences.

### 2.3. Quantification of Methylation

The data were analysed and the percentage of methylation at each of the dilutions was determined using the PyroMark^TM^ Q24 2.0.8 software (QIAGEN GmbH, Hilden, Germany).

## 3. Results

### 3.1. Different Assays Report Different Extent of Unequal Amplification of the Methylated and Non-Methylated Template

**Figure 1:**
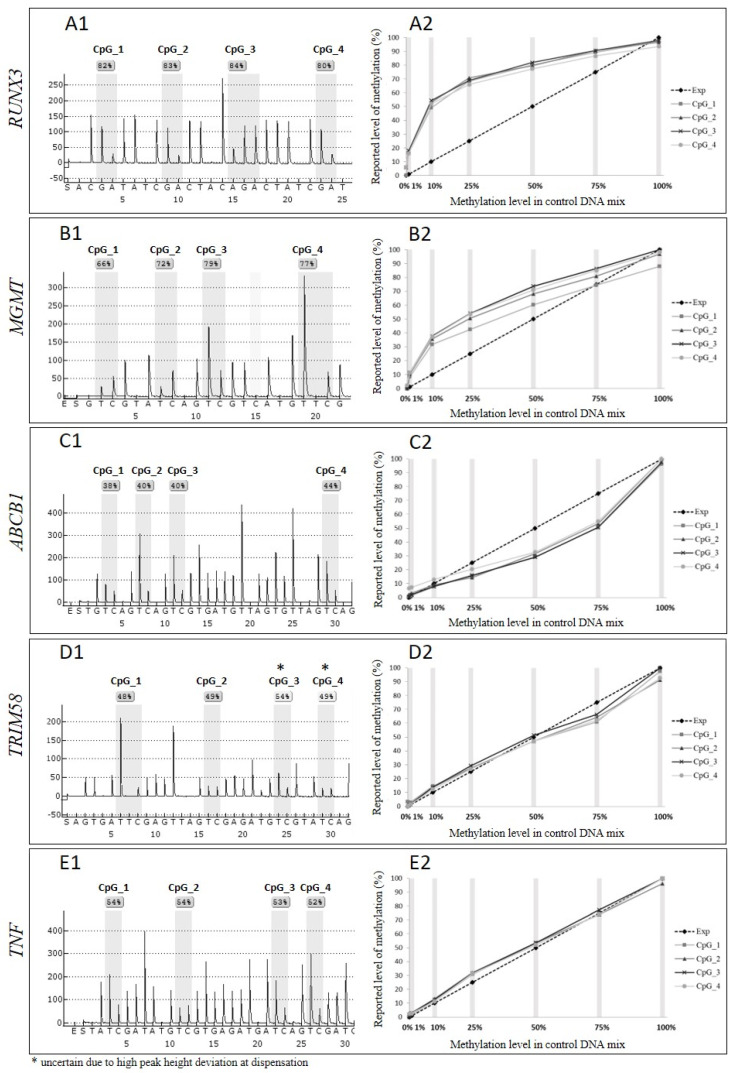
Comparison of the reported and expected methylation levels for pyrosequencing assays, including panel (**A1**,**A2**)—*UNX3* gene, (**B1**,**B2**)—*MGMT*, (**C1**,**C2**)—*ABCB1*, (**D1**,**D2**)—*TRIM58* and (**E1**,**E2**)—*TNF*. Panels denoted with 1 describe the reported methylation levels by pyrosequencing for the control mix with 50% methylated template in a non-methylated background. Panels denoted with 2 illustrate the correlation between the expected level of methylation (dashed line, “Exp”) and methylation level reported by pyrosequencing for each CpG site (1–4 lines in shades of grey) in each of the control mixes (grey vertical lines), including 0% (100% of non-methylated control template), 1%, 10%, 25%, 50%, 75% (of methylated template in non-methylated template), and 100% methylated template.

To initially assess the extent of unequal amplification of the loci targeted by four previously published and one commercially available pyrosequencing assay (Table 1), we mixed bisulphite-modified methylated and non-methylated control templates in equal proportions (50 and 50%) and evaluated the pyrograms obtained from PCR amplification of this control mix. As the target, loci contained varying numbers of the CpG sites, we focused this analysis on the first four CpG sites in each of the analysed sequences (Figure 1). The methylation levels reported by pyrosequencing for this control mix differed from the expected 50% methylation level for three of the assays. Specifically, the assay targeting *RUNX* reported 80–82% methylation (Figure 1 panel A1), *MGMT* 66–79% (Figure 1 panel B1), and *ABCB1* 38–44% (Figure 1 panel C1). For *TRIM58* (Figure 1 panel D1) and *TNF* (Figure 1 panel E1) the pyrosequencing correctly reported 50% methylation level.

### 3.2. Unequal Amplification Depends on the Proportion of Methylated to Non-Methylated Template

To investigate the influence of unequal amplification on the dynamic range of the pyrosequencing assays, we assessed the methylation level reported by pyrosequencing for a range of the control mixes with known proportions of methylated to non-methylated template. In Figure 1 panels: A2–E2, the dashed lines represent expected methylation levels that should be reported in each of the control mixes, assuming equal amplification of both templates and the methylation levels reported by pyrosequencing for each of the CpG sites in the control mixes are represented by four lines in shades of grey. The plots representing the reported data for each of the CpG sites methylation level overlap, indicating that a consistent number of the PCR product copies was sequenced throughout the amplicon. However, the reported methylation level for three out of five assays differs significantly from the expected levels. For the *RUNX3* assay (Figure 1 panel A2), the largest discrepancy between expected and reported methylation level was detected for 25% control mix, for which pyrosequencing reported almost 70% methylation. In the control mix containing 10% of methylated template, pyrosequencing reported over 50% methylation level. The smallest difference between expected and reported level of methylation was reported for 1% and 75% control mixes. For the *MGMT* assay we observed (Figure 1 panel B2) the most significant discrepancies between expected and reported methylation levels for 10% and 25% control mixes, with pyrosequencing reporting almost 30% and 50% methylation for those control mixes, respectively. Similar to the *RUNX3* and *MGMT* assays, the pyrosequencing reported the smallest differences between expected and reported level of methylation for 1% and 75% control mixes. The *ABCB1* assay (Figure 1 panel C2) underestimated the methylation levels in the control mixes with largest discrepancy between expected and reported levels of methylation observed for 75% control mix, for which pyrosequencing reported less than 50% methylation level, and for 50% control mix, the assay detected 30% methylation. The expected and reported levels of methylation for this assay did not differ for 1% and 10% control mixes. Two of the assays used in our experiments, the *TRIM58* and *TNF* assays (Figure 1 panel D2 and E2), reported expected methylation levels for each of the mixes of methylated and non-methylated templates.

### 3.3. Annealing Temperature Does Not Influence Unequal Amplification

**Figure 2:**
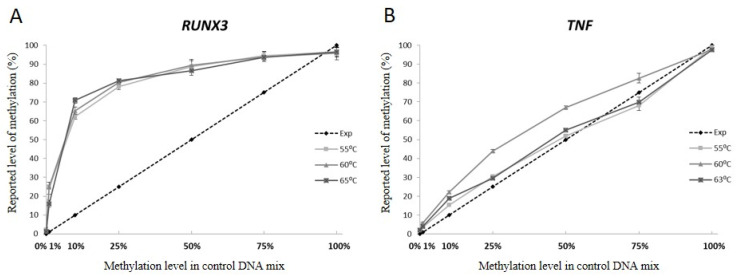
The influence of varying annealing temperatures on the quantitative methylation assessment by pyrosequencing of (**A**) *RUNX3* and (**B**) *TNF* genes sequences. The dashed line depicts expected methylation level assuming equal amplification of methylated and non-methylated versions of the template. Lines in shades of grey describe the methylation level reported by pyrosequencing data at three different annealing temperatures for each of the control mixes. The reported methylation level for each of the control mixes is an average level of methylation over the analysed CpG sites sequenced in the given amplicon.

The optimization of the PCR annealing temperature is the first step in the PCR assay development and correct annealing temperature is critical for the optimal efficiency of the PCR. To assess whether the optimization of the annealing temperature compensates for the unequal amplification, we performed amplifications of the *RUNX3* and *TNF* assays at three different annealing temperatures and again compared the expected and reported methylation levels. The curves in Figure 2 panels A and B show the methylation levels reported by pyrosequencing for each of the control mixes after amplification at three different annealing temperatures. The curves overlap for both of the assays used in the experiment, indicating that a change of the annealing temperature did not affect the unequal amplification.

## 4. Discussion

With the increasing number of new methylation biomarkers introduced into in vitro diagnostics (for comprehensive review of the methylation-based IVD tests please refer to: [10]), the demand for technologies enabling accurate methylation assessment is increasing. Although currently mainly qualitative assessment of methylation is utilized in diagnostic settings [11,12,13], quantitative or semiquantitative evaluation of methylation data is critical for the precise measurement of the methylation level, and also to establish a cut-off for the clinically relevant methylation level. The importance of the establishment of the clinically relevant level of methylation of a biomarker was recently highlighted by Hegi et al. [14] in a large-scale clinical study of the diagnostic utility of the *MGMT* gene methylation testing in patients with newly diagnosed glioblastoma (GBM). This study aimed to determine the methylation level, allowing the selection of patients for therapy omitting temozolomide (TMZ), while not excluding the patients who may benefit from the TMZ treatment. The results of the study indicated that some of the patients classified as non-methylated, according to the methylation level cut-off, in this study could benefit from the temozolomide treatment, and the lowest margin for the detection for the *MGMT* methylation testing used in this study needs to be reconsidered.

To establish a detection cut-off, semi-quantitative and quantitative methods are required and in the case of methylation detection with technologies that utilize the MIP primers unequal amplification of the template can make specific assays unsuitable for detection of methylated or non-methylated templates (non-methylated template in the case when the hypomethylation of the gene is a clinically significant event).

Pyrosequencing is one of the most frequently used technologies for quantitative methylation assessment in research and the number of applications of this technology in diagnostic settings is also increasing. Thus, a comprehensive assessment of the limitations of this technology is necessary.

In our study, two of the pyrosequencing assays showed linear correlation between expected and reported methylation levels and the performance of those assays was not affected by PCR conditions. At the same time, for three of the assays, we observed a significant over-amplification of the methylated or non-methylated template that significantly limited the dynamic range of the assays. The changes of the PCR conditions had no effect on the amplification of the methylated and non-methylated template. Most importantly, our results showed that the extent of unequal amplification differed between specific mixes of the templates with different proportions of the methylated to non-methylated template. This may in principle mean that for some pyrosequencing assays, the correlation between reported and expected methylation level is not linear. Consequently, for those assays, the dynamic range for measurement of the methylation in the sample may be difficult to establish.

In conclusion, our results indicate that when using methylation screening technologies such as pyrosequencing that rely on MIP primers, the extent of the unequal amplification of the methylated and non-methylated template needs to be assessed. In the case when over-amplification of one of the templates takes place, calculation of the methylation levels or cut-offs for a meaningful methylation level needs to be corrected for the values reflecting the overamplification. However, our results also indicate that a strategy to correct the methylation level calculation for a factor reflecting unequal amplification may not be feasible in some cases due to non-linear correlation between expected and detected methylation levels. We have not tested the influence of PCR bias on pyrosequencing-based assessment of methylation at the heterogeneously methylated locus. However, our (and other author’s) previously published work shows that the quantification of methylation at heterogeneously methylated locus is not possible without cloning and subsequent sequencing of single molecules of templates present in tested sample [15]. It is also worth mentioning here that a primer design strategy that we developed to overcome PCR bias can increase the sensitivity of the methylation detection by pyrosequencing [5].

## Figures and Tables

**Table 1:** Primer sequences, product lengths and other pyrosequencing characteristics for each assay.

Gene Name	Primer Sequence (Forward, Reverse, Sequencing)	T_a_ [°C]	PCR Product Length [bp]	Pyro Product Length (Analyzed/Total CpGs [No.])	Primers Ref.
***RUNX3***	5′-BIOTIN-AAGGGGTGATTTGTAGTGAAGTTTA-3′ 5′-CTCTACCAATCCAACCCCACTTCTTCT-3′ 5′-CCCACTTCTTCTTAAACC-3′	60	169	136 (4/14)	[6]
***MGMT***	Not disclosed	53	Not disclosed	Not disclosed(4/4)	*
***ABCB1***	5′-GTTGGAGGTGAGATTAATTTT-3′ 5′-BIOTIN-AAACCCCCAACTCTACCT-3′ 5′-GAGAGTAGTAAGAGGGA-3′	58	162	105 (4/8)	[7]
***TRIM58***	5′-TGTTYGGTGTGTTTGGATTTTTTGTAG-3′ 5′-BIOTIN-CACRCTCTCCACCAAACCC-3′ 5′-ATAGTTTTTGTTTTAGGT-3′	59	201	131 (4/17)	[8]
***TNF***	5′-GAGGTTAAGTTTTGGTATGAGTTTAT-3′ 5′-BIOTIN-CCTCCTCACAAAACAATAATCC-3′ 5′-AGTTGGAGAAGGGTG-3′	60	176	91 (4/6)	[9]

T_a_—annealing temperature, * therascreen^®^ MGMT Pyro^®^ Kit, Qiagen, Hilden, Germany.
